# Sphingolipid metabolism in brain insulin resistance and neurological diseases

**DOI:** 10.3389/fendo.2023.1243132

**Published:** 2023-10-06

**Authors:** Meng Mei, Maochang Liu, Yan Mei, Jing Zhao, Yang Li

**Affiliations:** ^1^ Department of Pharmacy, Wuhan Children’s Hospital (Wuhan Maternal and Child Healthcare Hospital), Tongji Medical College, Huazhong University of Science and Technology, Wuhan, China; ^2^ Administrative Office, Beijing University of Chinese Medicine, Beijing, China

**Keywords:** sphingolipid metabolism, brain insulin resistance, ceramide, sphingosine-1-phosphate, Alzheimer’s disease, Parkinson’s disease

## Abstract

Sphingolipids, as members of the large lipid family, are important components of plasma membrane. Sphingolipids participate in biological signal transduction to regulate various important physiological processes such as cell growth, apoptosis, senescence, and differentiation. Numerous studies have demonstrated that sphingolipids are strongly associated with glucose metabolism and insulin resistance. Insulin resistance, including peripheral insulin resistance and brain insulin resistance, is closely related to the occurrence and development of many metabolic diseases. In addition to metabolic diseases, like type 2 diabetes, brain insulin resistance is also involved in the progression of neurodegenerative diseases including Alzheimer’s disease and Parkinson’s disease. However, the specific mechanism of sphingolipids in brain insulin resistance has not been systematically summarized. This article reviews the involvement of sphingolipids in brain insulin resistance, highlighting the role and molecular biological mechanism of sphingolipid metabolism in cognitive dysfunctions and neuropathological abnormalities of the brain.

## 1 Introduction

Since the first actual sphingolipid was isolated from brain tissue in the late 19th century, the structure, metabolism and significance of sphingolipids in biological processes have been deeply explored. Through the continuous development of analytical tools and methods, the understanding of sphingolipids signal transduction has also been enhanced. These findings link sphingolipids to a large amount of human endocrine diseases, including obesity, diabetes, insulin resistance and neurodegenerative disorders (1, 2). Additionally, brain is rich in sphingolipids, and the pathophysiological processes of the brain are closely related to sphingolipids. Emerging evidence verified that the significant changes of sphingolipids and its metabolism were involved in various neurological diseases such as Alzheimer’s disease (AD), Parkinson’s disease (PD), amyotrophic lateral sclerosis, Gaucher disease, as well as Farber disease. However, the biochemical and molecular basis of the bioactive sphingolipids remains challenging, including the identification of molecular mechanisms, the study of lipid-protein interactions and the development of appropriate therapeutic strategies.

### 1.1 Diversity of sphingolipids

With the development of lipidomic technology, more than 600 sphingolipids have been detected in human beings (3). Sphingolipids are amphoteric lipids with a long-chain fatty acid at one end and a polar alcohol at the other end. In terms of structure, sphingosine is one of the main sphingosine bases, making it possible to distinguish sphingolipids from other lipids (4). Variations in the length of the terminal chain and the number as well as the position of functional groups (including double bonds, hydroxyl groups, etc.) can be combined with different polar head group arrangements to produce thousands of unique compounds (5). Among sphingolipids, the bioactive sphingolipids mainly include sphingosine, ceramide (Cer) and sphingosine-1-phosphate (S1P). In addition, sphingomyelin (SM), ceramide-1-phosphate (C1P), glucosylceramide, lactosylceramide, and certain gangliosides are also considered as bioactive sphingolipids candidates (4). The diversity of sphingolipids directly or indirectly contributes to the multiple biological functions of sphingolipids.

### 1.2 Metabolism and function of sphingolipids

Sphingolipids are commonly found in plant and animal membranes, and their primary function is to serve as basic structural components in eukaryotic membranes (6). In addition, sphingolipids are involved in regulating a variety of signaling pathways. Bioactive sphingolipids and their metabolites are important bioactive signaling molecules in mediating numerous biological functions, and participate in regulating various biological processes such as inflammation, apoptosis, cell cycle and metabolism (7, 8).

The lipid raft model was first proposed by K. Semons et al, which refers to the lipid bilayer plasma membrane, cholesterol (Ch) and sphingolipids form a relatively ordered lipid phase, like floating on the lipid bilayer “raft” (9). Sphingolipids contain long and saturated fat acyl chains. The arrangement of these chains is highly ordered with a large amount of Ch filling between their tight packing, forming a dense and Ch-enriched microdomain. The lipids that make up membrane rafts, including sphingolipids (glycosphingolipids and sphingomyelins) and Ch, are collectively referred to as raft lipids. Lipid rafts carry a variety of membrane proteins and serve as platforms for protein fixation to perform biological functions (10).

**Figure 1:**
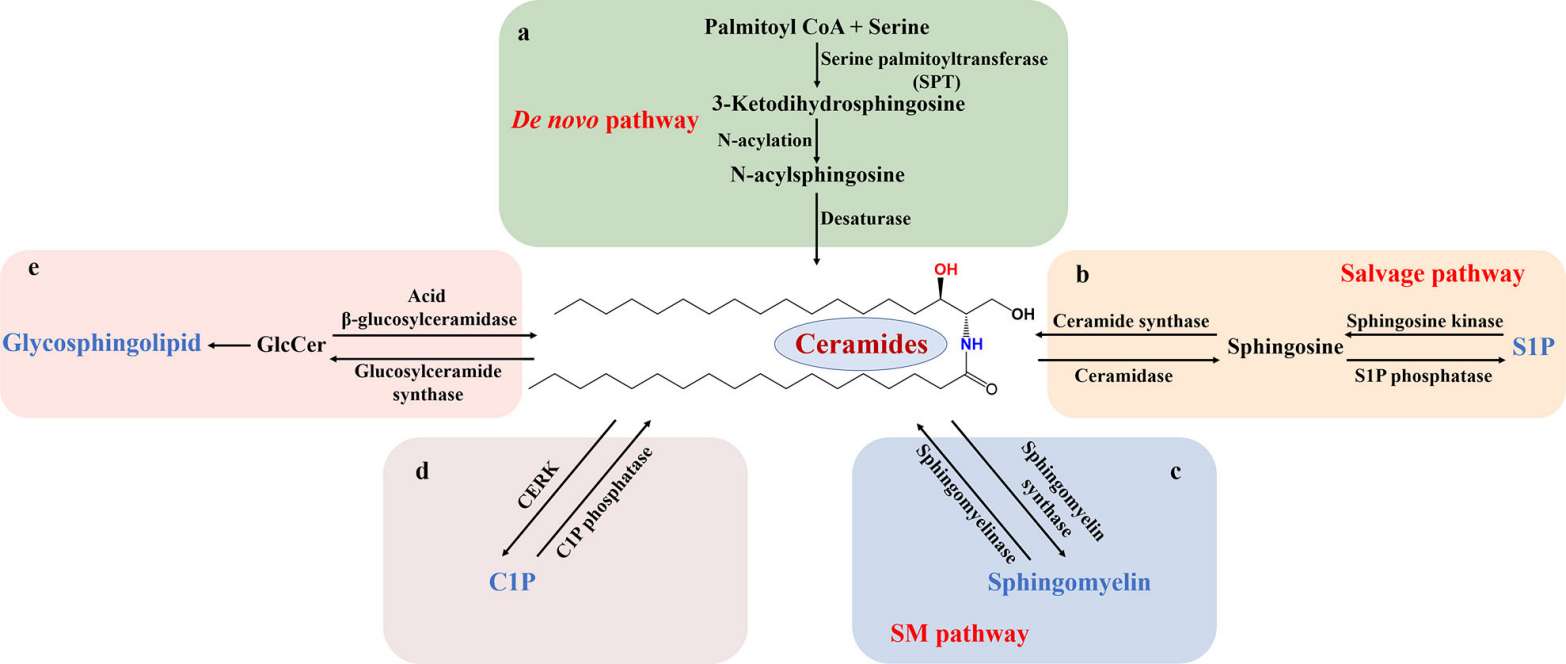
Schematic diagram of sphingolipid metabolism. Sphingolipid metabolism is divided into several chunks centered on Cer. **(A)** The *de novo* biosynthetic pathway is SPT-initiated action of serine and palmitoyl-CoA in the endoplasmic reticulum, followed by successive reactions that produce Cer. **(B)** In the salvage pathway, complex sphingolipids, such as S1P, is converted into Cer with the influence of sphingosine kinase and ceramide synthase. Correspondingly, Cer is deacylated to sphingosine, which is phosphorylated to S1P. **(C-E)** In the sphingolipid catabolic pathway, SM, C1P, and GlcCer are hydrolyzed, leading to Cer formation. S1P, sphingosine-1-phosphate; SM, sphingomyelin; C1P ceramide-1-phosphate; CERK, ceramide kinase; GlcCer, glucosylceramide.

The metabolic process of sphingolipids is divided into synthesis, degradation, transport (recycling) and utilization (mutual conversion) *in vivo* (11). With Cer as the metabolic center, the *de novo* synthesis of sphingolipids begins with serine and palmitoyl-coenzyme A (palmitoyl CoA). They are catalyzed and condensed by serine palmitoyl transferase (SPT) in the endoplasmic reticulum to form sphingosine bases (12). Further N-acylation by various fatty acyl-CoA enzymes to form N-acylsphingosine (dihydrosphingosine) is followed by desaturation, and thus produces Cer (13, 14) (**Figure 1A**). In addition to *de novo* synthesis, Cer is also remedied by converting complex sphingolipids (e.g. S1P, etc.) to sphingosine, and then formed by the action of ceramide synthase to N-acylate sphingosine, which is called salvage pathway. Sphingosine is phosphorylated by sphingosine kinase (SK) to form sphingosine-1-phosphate (**Figure 1B**). Subsequently, S1P is irreversibly cleaved to phosphorylethanolamine and cetylaldehyde by sphingosine-1-phosphate lyase. The direct hydrolysis of SM can also produce Cer (15). Cer can be metabolized into more complex sphingolipids by the addition of head groups. For example, Cer is connected with phosphocholine or carbohydrate head to form sphingomyelins or glycosphingolipids, respectively. Moreover, Cer is phosphorylated to ceramide-1-phosphate (C1P) by ceramide kinase (CERK) or hydrolyzed to sphingosine by ceramidase (**Figures 1C–E**). Sphingolipid metabolism varies depending on the organism. For example, there are at least five different ceramidases and five different sphingomyelinases (SMase) located in the plasma membrane, organelles (endoplasmic reticulum, golgi apparatus, nucleus, lysosome and mitochondria) and extracellular space. Studies have shown that about 40 enzymes in mammals are involved in the regulation of this series of biological metabolic processes (4).

The diversity of sphingolipid metabolism determines that defects in sphingolipids synthesis and decomposition are associated with various human diseases and pathological conditions. For example, Cer promotes the occurrence and development of PD by regulating mitochondrial autophagy (16, 17). The production of sphingosine and/or Cer may mediate cancer cell death by activating *de novo* synthesis pathways, SM hydrolysis, and salvage pathway (18, 19). Furthermore, SM is identified as one of the biomarkers of the recurrence and death after myocardial infarction. Reducing Cer levels promotes cell survival and thus provides cardiac protection after myocardial infarction (20, 21). In the future, the link between sphingolipid and various diseases and the corresponding therapeutic targets will continue to be discovered and supplemented.

### 1.3 Intracerebral sphingolipids

Central nervous system contains lipids such as phospholipids, sphingolipids and cholesterol. Sphingolipids are especially abundant in central nervous system compared with other parts. The central nervous system mainly consists of neurons and glial cells. Sphingolipids play different signaling roles in different cells due to the influence of enzymes and proteins presented in the local environment. Similarly, even if the same lipid is produced in the plasma membrane, endoplasmic reticulum, lysosome or mitochondria, it also exerts different biological effects (22).

#### 1.3.1 Neuron

Neurons are the most basic structure and function unit of central nervous system. Lipids play an important role in the structure and function of neurons. Sphingolipids are not only related to the development of neurons, but also affect the secretion of neuronal exosomes, thus influencing the conduction of nerve impulses. The sphingolipids metabolic pathway of dopaminergic neurons in the substantia nigra is involved in neuroimmunoinflammation and neurodegeneration in PD. The dopamine neuron-specific translation group showed that sphingolipids synthesis pathway was significantly down-regulated in MPTP treated mice (23), which indicated that the destruction of sphingolipid metabolism could lead to significant neuronal damage. Sphingolipid metabolism is compartmentalized, so its metabolism in different organelles may affect the function of neurons through different pathways. Studies showed that imbalances in lipids (including Cer and sphingolipid intermediates) led to mitochondrial and endolysosome dysfunction, thus resulting in neuron death (11). Kohei Yuyama et al. found that neuronal exosome secretion was regulated by sphingolipids metabolizing enzymes including neutral sphingomyelinase and sphingomyelin synthase (SMS) (24). Both S1P and Cer are called “bioactive sphingolipids”, but their functions are antagonistic in many ways. In central nervous system, Cer and S1P are involved in the regulation of neuronal survival and death, therefore participating in physiological and pathological processes such as aging, AD and PD (25).

#### 1.3.2 Microglia

A century ago, Spanish neuroscientists first proposed that microglia were a unique group of cells in central nervous system. Microglia play an extremely crucial role in the physiological process of the central nervous system as the intrinsic immune-effector cells (26). Similar to the properties of macrophages, microglia are a double-edged sword in disease process. On the one hand, microglia release a large amount of pro-inflammatory factors and neurotoxic substances, which aggravates the injury. On the other hand, through the phagocytosis function and secretion of neurotrophic factors, microglia promote nerve repair. Active sphingolipid metabolites lead to neuroinflammation and activate microglia. For example, S1P activates the volume-regulated anion channels on microglia that controls ATP release, which is an important inflammatory mediator in neuropathic pain (27). The role of microglia in regulating the neurodegeneration of AD and PD has been well studied. Researchers pointed out that the lipid metabolism of microglia reflected the sensitivity of the brain to amyloid accumulation (28). Genetic studies have shown that the genetic risk of PD is enriched in sphingolipid-related factors (29). Among them, glucocerebrosidase β1 (GBA1) has been widely concerned as one of the biggest risk factors for PD (30). Notably, recent single-cell maps of mouse brains showed that most risk genes, including GBA1, are expressed by microglia rather than neurons (31). Chronic α-synuclein dysfunction in PD, dementia with Lewy bodies, and multiple system atrophy can be caused by microglia’s pathological modification on α-synuclein, which is mediated by unbalanced sphingolipid metabolism (28).

#### 1.3.3 Astrocyte

Astrocytes are essential support cells in the brain and secrete neurotrophic factors, which regulate synaptogenesis, neuronal differentiation and neuronal survival. These biological functions determine that astrocytes are an important link in the occurrence and development of neurological diseases (32). However, the mechanisms involved in these processes have not been fully illustrated. Proinflammatory stimulation-induced disordered sphingolipid metabolism affects the metabolism of astroglia. Researchers established experimental models of autoimmune encephalomyelitis and multiple sclerosis using non-obese diabetic mice. Further, through gene expression and knockout analysis, it was confirmed that lactosylceramide (LacCer) in astrocytes could exacerbate the occurrence and development of experimental autoimmune encephalomyelitis in non-obese diabetic mice through biosynthetic pathway. It has also been demonstrated that sphingomyelin metabolism could enhance the pathogenicity of astrocytes in a variety of neuropathic diseases by activating the nuclear factor-kappa B pathway (33). Additionally, modulation or elimination of S1P receptor 1 (S1PR1) in astrocytes could promote neuronal survival and relieve the pathological injury of experimental autoimmune encephalomyelitis mice (34, 35).

### 1.4 Mechanism of sphingolipids causing nervous system diseases

The most characteristic pathology of AD is neurofibrillary tangles formed by deposition of amyloid beta (Aβ) and phosphorylated microtubule-associated protein Tau in extracellular amyloid plaques. Cer promotes Aβ formation by affecting the cleavage of the transmembrane protein amyloid precursor protein (36, 37). In mitochondria, increased Cer impaired oxidative phosphorylation, disrupted membrane potential, and facilitated the release of pro-apoptotic proteins into the cytoplasm (38–41). Organelles with Cer-rich membranes, such as endoplasmic reticulum and endosomes, could also cause neuronal and glial damage due to oxidative stress or damaged proteostasis, which led to the accumulation of neurotoxic peptides or proteins (e.g. Aβ, tau, synuclein and Huntington protein). Exposure of hippocampal neurons to Aβ induced membrane oxidative stress and accumulation of Cer and cholesterol, whereas treatment with alpha-tocopherol or sphingomyelin synthesis inhibitors prevented lipid overload, thereby protecting them from Aβ-induced death. These findings suggest that membrane-related oxidative stress induced by Aβ in the pathogenesis of AD may lead to the dysfunction of Cer and cholesterol metabolism, thereby triggering a cascade of neurological insults (42).

Autophagy-lysosomal pathway (ALP) is essential for the survival of undivided neurons and the maintenance of nervous system homeostasis through clearance of abnormal protein aggregates and damaged organelles. The acidic cavity of lysosome contains a large number of hydrolases, including proteases, lipases and nucleases (43). The metabolism of glycosphingolipids (GSLs) to Cer mainly occurs in lysosomes. When the hydrolases are insufficient in this process, it can lead to the accumulation of corresponding substrate. Since brain neurons are particularly sensitive to lipid accumulation, lysosomal lipid storage disorders, such as sphingolipids and ganglioside deposition, often affect the brain. Some specific type of lysosomal lipid storage disorders were associated with altered amyloid precursor protein and tau metabolism, which was also observed in AD (44). Additionally, excessive phosphorylation of tau protein also contributed to the instability of neuronal microtubules and triggered autophagy dysfunction, which affected the location and function of lysosomes. Amyloid precursor protein can be cleaved through non-amyloid or amyloid pathways. If lysosome function is impaired or Aβ production is increased, Aβ will accumulate in neurons, leading to cell death and thus causing pathological response of AD. Yumiko V Taguchi et al. have shown that glucosyl sphingosine acts as an inducer of endogenous α⁃ synuclein aggregation in human and mammalian neurons (45). Therefore, targeting acid ceramidase 1 and glucocerebrosidase 2, which regulate the production and metabolism of glucosylsphingosine, holds a promising prospect for the treatment of mutant GBA-associated PD.

Aside from neurodegenerative diseases, sphingolipids also play an important role in other neurological diseases. Cer chronically inhibited ionotropic glutamate receptor-mediated synaptic transmission in hippocampus and its mechanism might possibly due to the activation of postsynaptic protein phosphatase, which was one of the common mechanisms between Cer-induced persistent depression and long-term depression (46). Furthermore, S1P expression in astrocytes has been shown to influence the severity of experimental autoimmune encephalomyelitis, and relevant data suggested that the S1P signaling pathway in the CNS was a target for MS therapy (35). The S1P-related signaling pathway was divided into two main modes of action: extracellular and intracellular mechanism. After the production of S1P, it acted on the cell surface receptor through autocrine or paracrine, thus producing cell proliferation, migration, inflammatory cytokine infiltration, autoimmunity and other extracellular effects. It could also exercise the second messenger function in the cell, directly acting on the intracellular target to regulate gene transcription, protein modification and Ca^2+^ level. The S1P analogue, fingolimod (FTY720), is the first new immunosuppressant to be used orally for the treatment of MS. As a prodrug of sphingosine, FTY720 did not directly inhibit the activation and proliferation of lymphocytes. Instead, fingolimod was phosphorylated by SphK1 into active metabolite p-FTY720, which bound to specific S1P receptors except S1PR2, affected chemokine-mediated lymphocyte homing, reduced the number of peripheral blood lymphocytes and inhibited lymphocyte activity, thus played an immunosuppressive role (47). Meanwhile, FTY720 easily crosses the blood-brain barrier due to its lipophilicity. When acting on the endothelial cells of blood-brain barrier, it could reduce cell death induced by inflammatory cytokines. FTY720 significantly down-regulated the release of lipopolysaccharids-stimulated microglia inflammatory mediators, thereby alleviating neuroinflammation (48). In addition, FTY720 was able to reduce sphingosine-1-phosphate lyase, thereby increasing S1P concentrations in the brain, which was consistent with a significant reversal of neurological deficits (49).

## 2 Association between brain insulin resistance and disease

ATP is the main form of energy in brain, and about 95% of ATP is produced by oxidative phosphorylation of glucose in the mitochondria and supplemented by aerobic glycolysis in the cytoplasm (50). Glucose passes through the blood-brain barrier by proteins in the family of sodium-dependent glucose cotransporters (SGLT1 and SGLT2) and the sodium-independent glucose transporters (GLUTS) (51). Neurons have the highest demand for energy and require a constant supply of glucose, so the ability to transport glucose to the brain exceeds the brain’s energy requirements by two to three times under normal conditions (52). Increasing evidence showed that impaired brain energy metabolism was involved in the progression of neurodegenerative diseases in the brain, and the low glucose metabolism in the brain of patients with central nervous system diseases has multiple causes, these include, but are not limited to the reduced glucose uptake by neurons, abnormal tricarboxylic acid circulation and glycolysis, loss of energy support by glia to neurons, and brain sugar processing problems caused by brain insulin resistance (53–55). Literatures suggested that insulin signaling regulates synaptic plasticity, neurogenesis, and glucose metabolism in the brain, thereby promoting learning and memory (56).

### 2.1 Brain insulin resistance

Insulin is a peptide hormone secreted primarily by pancreatic β cells and is known to regulate glucose metabolism in peripheral tissues. Insulin receptors, which are expressed early in the ontogenetic development, play an important role in regulating the metabolism of carbohydrates, lipids as well as proteins and controlling the level of brain neurotransmitters. The dysfunction of insulin receptors is associated with a variety of diseases (57).

Brain is one of the insulin-sensitive tissues, and insulin receptors are distributed in most cells of the brain. The highest density of insulin receptors is found in the hypothalamus, olfactory bulb, hippocampus, striatum, cerebral cortex and cerebellum. Evidence suggested that cerebral insulin regulated energy homeostasis by coordinating nutrient distribution (58). However, it is worth noting that a significant number of people have reduced or even nonexistent brain response to insulin, which is a condition known as cerebral insulin resistance (also named brain insulin resistance, BIR) (59). Insulin resistance is generally defined as reduced sensitivity of target tissues to insulin action or altered biological response of target tissues to insulin stimulation, resulting in reduced or loss of insulin-stimulated glucose uptake and utilization (60). Peripheral metabolic changes led to neuroinflammation and insulin resistance in the brain, which resulted in defects in neuronal signaling and synaptic plasticity (61). Correspondingly, persistent peripheral insulin resistance could also cause misalignment of signal transduction in the brain insulin receptors, leading to the development of BIR. Brain sugar processing problems caused by BIR are also known as “hypoglycemia”, where brain cells do not respond to insulin as they normally would. This means that brain cells do not have enough insulin to burn glucose, and glucose metabolism becomes increasingly sluggish, which often impairs the brain’s synaptic, metabolic, and immune response functions. Thus, BIR may increase the risk of neuroinflammation and neurodegeneration. These brain cells, which lack the ability to metabolize glucose, decline, thus leading to symptoms of a variety of diseases, including diabetes, cancer and neurodegenerative diseases such as AD and PD.

### 2.2 Brain insulin resistance is involved in a variety of diseases

#### 2.2.1 Advances in brain insulin resistance and type 2 diabetes mellitus

Diabetes mellitus (DM) is one of the most common metabolic diseases. In 2021, about 537 million people aged 20 to 79 have diabetes. The number is expected to grow to 783 million by 2045. There are two types of diabetes depending on the etiology. Type 1 diabetes mellitus is caused by genetic or autoimmune destruction of insulin-producing islet beta cells and accounts for 5-10% of total diabetes cases. Correspondingly, type 2 diabetes (T2 DM) accounts for about 90% of all diabetes cases and is mainly characterized by insulin resistance (62, 63). Neuroimaging studies have shown that the brain structure and function of patients with T2 DM are different from those of healthy people. However, there is no evidence to prove that T2 DM related cognitive impairment and neuroimaging changes are the results of BIR (64, 65). Nevertheless, existing animal studies suggested that T2 DM was associated with systemic and cerebral insulin resistance. For example, all genetic models of T2 DM, pharmacologically induced models, and high-fat diets rodents exhibited systemic insulin resistance, hyperglycemia, and cerebral insulin resistance, as well as other brain defects such as synaptic abnormalities (structural, molecular, and neurophysiological) and memory deficits (66, 67). There have also been some human studies suggested that intranasal insulin administration could normalize cerebral hemispheric connectivity, improve local cerebral perfusion and enhance cognitive performance in T2 DM patients with cognitive dysfunction (68, 69). These findings indicate that cerebral insulin resistance may be overcome with higher doses of insulin to improve systemic or cerebral impaired function in patients with T2 DM.

#### 2.2.2 Brain insulin resistance and Alzheimer’s disease

The molecular signaling pathway of insulin also plays a role in regulating synaptic neurotransmission, neuronal and glial metabolism, and neuroinflammatory responses in the brain. AD and type 2 diabetes share the similar pathogenic mechanisms such as inflammation, oxidative stress, dyslipidemia, impaired mitochondrial and synaptic function, and impaired insulin signaling in the brain (70). It has been reported that compared with healthy people, patients with AD have reduced or mislocated (not located on the membrane surface) insulin receptors in the brain and the affinity of insulin receptors is likely to decrease (71). Insulin directly affected the pathologic progression of AD by interacting with Aβ peptide. Co-localization of neurofibrillary tangles and brain markers of insulin resistance has also been shown in neuropathologic studies in patients with AD (72). In addition to increasing Aβ accumulation and promoting the formation of Aβ oligomers and neurofibrillary tangles, BIR also caused a surge in the activity of glycogen synthetase kinase-3β (73), phosphorylate tubulin associated unit excessively (74), thus became direct contributor to the progress of AD pathology. These evidences suggest that BIR is indeed involved in the development and progression of AD by affecting glucose metabolism. In this way, the level of glucose metabolism in the brain can be served as an early biomarker of AD.

#### 2.2.3 Brain insulin resistance and Parkinson’s disease

PD is often characterized by static tremor, decreased voluntary movement, increased stiffness, slow movement and postural instability, which often overlaps with AD, and the incidence of PD is second only to AD among neurodegenerative diseases (75). It has been mentioned above that the course of AD is related to BIR, whether the same situation also occurs in PD? BIR affected mesencephalon cortex circuits, including the prefrontal cortex, striatum, and hippocampus, which were participants of the major dopamine pathways that regulate energy-related behaviors. Studies have shown that BIR is associated with α-synuclein changes and dopamine loss in brain regions, and further corresponds to classic motor symptoms of PD (76). It has been reported that the insulin receptor mRNA in the substantia nigra of PD patients was significantly reduced compared to the control group of the same age (77). Andre Kleinridders et al. found that, after knocking out neuron-specific insulin receptors, the activities of monoamine oxidase and mitochondria in mice were impaired, and dopamine reuptake was enhanced (78). Perruolo et al. established a PED/PEA-15 overexpression animal model with a low dopaminergic phenotype and decreased activity, which was similar to Parkinson-like symptoms. The hypokinetic behavior of these animals might be due to the BIR and the depletion of dopamine in the striatum (79). Furthermore, studies have shown that BIR is associated with mitochondrial membrane potential depolarization, mitochondrial biogenesis impairment and ROS increase, and mitochondrial dysfunction is the most basic feature of PD pathogenesis (80, 81).

### 2.3 Sphingolipid metabolism in AD and PD

Abnormal sphingolipid metabolism has been shown to play a key role in the pathogenesis of neurodegenerative diseases including AD and PD. Timothy A Couttas et al. ‘s study showed that S1P decreased with the increase of Braak stage in brain regions with AD lesions, which demonstrates the loss of neuroprotective factor S1P in the pathogenesis of AD (82). In the early stages of AD, multiple glucoside deficiencies and elevated Cer were observed in the lesion area (42, 83). Changes in sphingolipid metabolism are also detected in plasma samples. Lipidomics showed that serum sphingolipids was reduced in AD patients compared to the healthy controls, and accompanied by the increased Cer levels (84). This claim is further validated by sphingomic studies demonstrating similar sphingolipid changes in the plasma of AD patients (85, 86). Additionally, several studies also revealed that gangliosides, especially GM 2 and GM 3, were up-regulated in AD model mice (87–90), while gangliosides GD1 was down-regulated (87). Despite the direct sphingolipid type changes, corresponding alterations in various sphingolipid metabolic enzymes have also been studied, such as the elevated expression of serine palmitoyl transferase and Cer synthase 1, 2 and 6 in the cortex, hippocampus, caudate nucleus and putamina nucleus (91). The sphingomyelinases of frontotemporal region and glucosyl ceramidase in cortices also showed an upward trend (92, 93).

An untargeted metabolomic and proteomic analysis of 36 PD patients and healthy people revealed that 83 metabolites were differentially expressed, of which 63% were fats and lipid molecules, and sphingolipids accounted for 25% of these lipid molecules. Further analysis of related metabolic pathways of the differentially expressed metabolites showed that only sphingolipids metabolic pathways were significantly enriched, while 6 metabolites involved in sphingolipid metabolism were significantly increased, suggesting that sphingolipids metabolic activation might be one of the potential pathogenesis of PD (94). In another multiracial cohort study of PD patients and healthy individuals, about 1,000 lipids were analyzed in the serum of each volunteer. Among them, the main lipids that distinguish PD patients/LRRK2 mutation carriers from healthy subjects included Cer, triglycerides, SM, etc., and significant changes in triglycerides and SM were also reflected in cerebrospinal fluid. The KEGG compound/pathway database is used to determine whether the target lipids are enriched in certain pathways. Results showed that sphingolipid metabolism, insulin signaling and mitochondrial function were the main metabolic pathways of dysregulation in PD. More interestingly, Cer and TG were mainly enriched in glycogen production, glucose transporter type 4 (GLUT4) translocation and glucose uptake pathway, which were also sub-pathway in the “insulin resistance” pathway (**Table 1**) (95).

**Table 1:** The sphingolipids in BIR and neurological disease.

Sphingolipids	Influence in BIR	AD	PD
Ceramide	1. Inhibit insulin signaling pathway (PI3K-AKT/GLUT4/IRS tyrosine phosphorylation/IKK-NF-κB) (95, 100, 104, 105) 2. Induce mitochondrial damage and lead to impaired glucose utilization (106) 3. Induce endoplasmic reticulum stress (107, 108)	**↑ (** 42)	**↑ (** 94, 95)
		Cause oxidative stress and impair protein homeostasis, leading to neuronal and glial damage Affect the cleavage of transmembrane protein amyloid precursor protein and promote the formation of Aβ (42, 109)	
Sphingomyelin	1. Inhibit the activity of γ-secretase 2. Reduce the accumulation of Aβ 3. Weaken sphingomyelin enzyme activity, reduce Cer, and play a protective role	**↓ (** 92)	**↑ (** 17, 98)
		Lysosomal sphingomyelin storage disorders, associated with altered metabolism of amyloid precursor protein and tau (44)	
S1P	1. Increase phosphorylated PI3K-AKT protein level (97) 2. Increase the ability of insulin resistant cells to take up glucose	**↓ (** 92)	* ( 25)
		S1PR1: reduce α-synaptic protein aggregation and increase brain-derived neurotrophic factor (110)	
Ganglioside GM1/GM3	1. Inhibit the activation of IRS-1 2. GM3 causes insulin receptors to separate from caveolin-1 (111)	**↑ (** 112)	**↓ (** 113)
		The depletion of GBA promotes the transfer of alpha-synuclein copolymers (45)	

* indicates a lack of clinical data on S1P in PD. PI3K-AKT, phosphatidylinositol-3-kinase-protein kinase B signaling pathway; GLUT4, glucose transporter type 4; IRS, insulin receptor substrate; IKK-NF-κB, IκB kinase-nuclear factor kappa B; Aβ, Amyloid beta; GBA, glucocerebrosidase. ↑: upregulate ↓: downregulate

As previously mentioned, S1P acts as an autocrine or paracrine messenger. Sphingosine kinases, which are mainly divided into Sphk1 and Sphk2, are biological enzymes responsible for S1P synthesis and regulation of bioactive sphingosine lipid homeostasis. Sphingosine kinase 2 is significantly down-regulated in the substantia nigra region of the midbrain of MPTP induced mice PD (96). It has also been found that inhibition of Sphk1 in PD model will further aggravate caspase-dependent apoptosis, thus causing the death of dopaminergic neurons (97). The most abundant sphingolipid in eukaryotic cells and plasma is SM. SM locates in the endoplasmic reticulum, Golgi apparatus, and nucleus is metabolized by neutral SMase1. In the mesencephalon of MPTP-induced PD mice, neutral SMase1 was reduced and SM was accumulated (98). The role of SM accumulation in the pathogenesis of PD may be multifaceted and related to mitochondrial dysfunction, inflammation, and α⁃ synuclein aggregation (17, 98).

### 2.4 Mechanisms of brain insulin resistance

The possible mechanisms of BIR are downregulated expression of insulin receptor, defective binding of insulin to its receptor and obstruction of insulin signaling pathway. These are often the results of multiple factors, including but not limited to age, genetic background, blood glucose levels, obesity, inflammation, and sensitivity to BBB transporters. Chronic hyperinsulinemia with overnutrition activated the mTOR/S6 kinase pathway and enhanced phosphorylation of insulin receptor substrate 1 (IRS-1) serine to induce hypothalamic insulin resistance (99). Various phosphatases are also involved in the regulation of insulin sensitivity. Protein phosphatase 1B and T-cell protein tyrosine phosphatase were involved in insulin signaling in the brain by dephosphorylating tyrosine residues of insulin receptors (100). Sphingolipids, which are involved in various physiological functions in the central nervous system, can also mediate the occurrence of BIR. Lipid-induced insulin resistance was described as early as 1941. After intravenous infusion of lipids, rabbits were found to be insensitive to insulin-induced hypoglycemia (101). Subsequently, it has been shown that neurodegeneration of cerebral insulin resistance can be mediated by neurotoxic Cer, which is easy to cross the BBB (102).

At present, a large number of studies have reported the relationship between elevated circulating fatty acids and insulin resistance. The increase in fatty acid lipids results in the accumulation of lipid droplets in various organs and tissues. In this way, sphingolipids such as Cer and other lipid intermediates are also formed successively (103). These bioactive lipids are associated with the pathogenesis of insulin resistance. However, the mechanism of sphingolipids, such as Cer and sphingomyelin, participating in BIR has not been systematically summarized yet.

## 3 Sphingolipid metabolism involved in brain insulin resistance

Previous studies have shown that insulin and glucose concentrations in peripheral blood receive and respond to central insulin signals through the pancreatic axis (114). Conversely, peripheral insulin resistance induced by high sugar leads to accumulation of insulin, which results in hyperinsulinemia. Then, hyperinsulinemia causes damage to target organs, including the brain. The main reason for this consequence is that insulin in the peripheral blood is actively transported into the brain. Short-term brain exposure to insulin has a positive effect on neuronal metabolism. However, prolonged exposure may cause hyperinsulinemia in the brain, which induces insulin resistance in central system neurons and increases the risk of developing neurological disease. Related studies have found that peripheral hyperinsulinemia contributes to increased insulin levels in the brain and further leads to insulin resistance in neurons (115). Based on the above, we should recognize the bidirectional relationship between peripheral insulin resistance and BIR. That is, peripheral insulin resistance can disrupt brain insulin activity and vice versa, thus leading to a vicious cycle (116).

### 3.1 Ceramide in BIR

Cer is mainly distributed in cell membranes and is the product of *de novo* biosynthesis or degradation of sphingolipids, as well as the precursors of a variety of sphingolipids. Cer and its related molecules are key components involved in the pathogenesis of BIR. They can be produced in muscle, liver, adipose tissue and brain. In skeletal muscle, Cer reduced AKT activity via protein kinase Cζ (PKCζ) and protein phosphatase 2A, leading to skeletal muscle insulin resistance (101). When obese mice induced by a high-fat diet were injected with myriocin, an inhibitor of the Cer *de novo* synthesis pathway, it could promote the phosphorylation of AKT in liver and muscle, sensitize insulin signaling, and promote glucose utilization and balance in the body (117). In this way, researchers indirectly demonstrate the role of Cer in insulin resistance.

**Figure 2:**
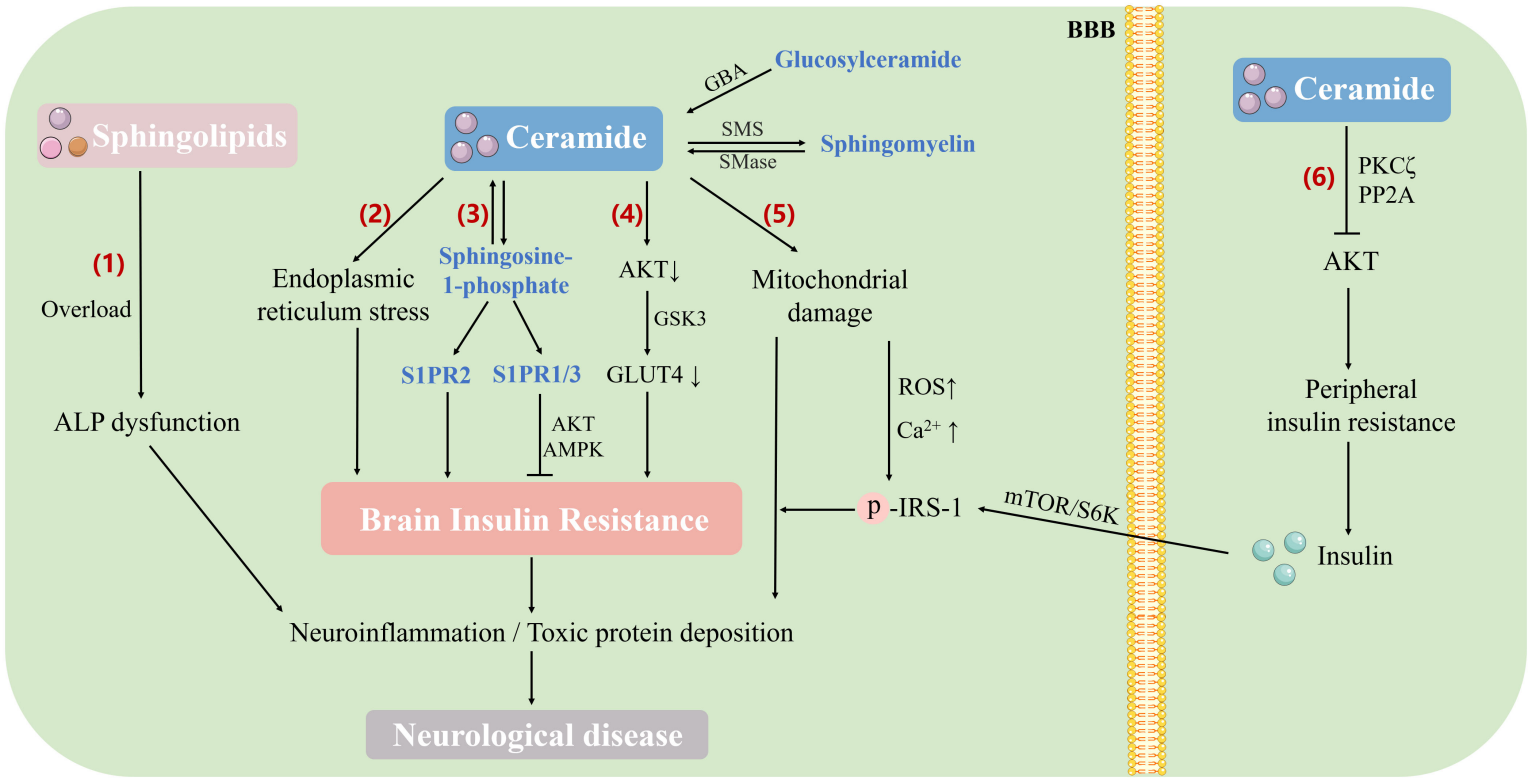
The mechanism of sphingolipid metabolism involved in brain insulin resistance and neurological diseases. Sphingolipids induce brain insulin resistance through different pathways, which directly or indirectly leads to neurological diseases (1). Accumulation of sphingolipids (such as Cer, ganglioside, and sphingomyelin) in the brain leads to lysosomal lipid storage disorders and dysfunction of the ALP, thus affecting amyloid precursor protein and tau metabolism (2). Excess saturated fatty acids and other sphingolipids in tissues promote Cer formation, induce endoplasmic reticulum stress, and further lead to insulin resistance (3). S1P antagonizes brain insulin resistance by activating the insulin signaling pathway (AKT/AMPK) through the receptor subtypes S1PR1 and S1PR3, while receptor subtype S1PR2 activation results in BIR (4). Excess Cer in the brain antagonizes the activation of AKT, inhibits the translocation of GLUT4 to the plasma membrane, affects glucose uptake, and causes brain insulin resistance (5). Cer induces mitochondrial dysfunction and ROS formation, which contributes to increased oxidative damage to mitochondrial DNA and impaired electron transport chain function, resulting in impaired Ca^2+^ processing capacity. Intracellular Ca^2+^ elevation phosphorylates insulin receptors and insulin resistance substrates, thus leading to brain insulin resistance (6). Elevated peripheral Cer reduces AKT activity through PKC ζ and protein phosphase 2A, leading to peripheral insulin resistance. Insulin crosses the BBB and causes chronic hyperinsulinemia, which in turn activates the mTOR/S6 kinase pathway, enhances the phosphorylation of IRS-1 serine and ultimately induces hypothalamic insulin resistance. ALP, autophagy-lysosomal pathway; AKT, protein kinase B; AMPK, adenosine 5’-monophosphate (AMP)-activated protein kinase; GSK3, glycogen synthase kinase-3; GLUT4, glucose transporter type 4; GBA, glucocerebrosidase; SMS, sphingomyelin synthase; SMase, sphingomyelinase; PKC ζ, protein kinase C ζ; PP2A, protein phosphase 2A; BBB, blood-brain barrier; IRS, insulin receptor substrate; mTOR/S6K, mammalian target of rapamycin S6 kinase pathway.

At the same time, Cer is liposoluble and easy to cross the BBB, causing cerebral insulin resistance (102). Increased Cer in the central nervous system antagonized insulin signaling via AKT pathway. When insulin bound to its receptor, tyrosine residues in the insulin receptor substrate (IRS) were phosphorylated, thus triggering a number of signaling cascades through activated kinases (118). Phosphorylation at the IRS tyrosine site activated the phosphoinositide 3-kinase-AKT (PI3K-AKT) pathway, while phosphorylation at the serine site inhibited it (105). After AKT activation, the critical molecule GLUT4 in the downstream pathway translocated to the plasma membrane for glucose uptake in neurons of the cerebral cortex, hippocampus, and cerebellum (119). When there was an excess of Cer in the brain, this pathway could be antagonized. Long-term elevation of Cer in tissues led to sustained impairment of metabolic homeostasis, driving insulin resistance and such kind of damage might be irreversible (120, 121). Excess saturated fatty acids in tissues mediated abnormal Cer formation, which further induced endoplasmic reticulum stress, mitochondrial dysfunction and ROS formation, while hypoxia-induced dyshomeostasis of Ca^2+^ could also be attributed to increased *de novo* lipid synthesis and accumulation of saturated fatty acids. Elevated intracellular Ca^2+^ phosphorylated insulin receptors and substrates of insulin resistance by activating Ca^2+^-dependent serine/threonine protein kinases, leading to the development of insulin resistance (121) (**Figure 2**).

### 3.2 Sphingosine-1-phosphate in BIR

S1P is a multifunctional lipid mediator involved in physiological processes including cell survival, neovascularization, inflammation, as well as neuronal development. Existing studies have shown that S1P is associated with important intracellular targets related to cancer, diabetes and neurological diseases. S1P possesses the capability to interconvert with Cer. Ceramidase catalyzes the deacylation of Cer to sphingosine, and SK phosphorylates the generated sphingosine base to S1P. S1P acts directly on intracellular targets as a second messenger. Besides, it can also be transported to extracellular by transporters and bind to corresponding S1P receptors to exert biological effects. The receptors mainly including five G-protein-coupled receptors, named S1PR 1-5 (122). Among them, S1PR1, S1PR2 and S1PR3 are mainly expressed in the immune, cardiovascular and central nervous system, which are related to hypertension, PD and other diseases (123). In mice, the absence of the S1P vector (Apolipoprotein M, ApoM) was associated with worsening insulin resistance. Similarly, serum ApoM levels were lower in T2 DM patients and negatively correlated with insulin resistance. *In vitro* and *in vivo*, the inhibition effect of S1P on insulin signaling was eliminated in the presence of the S1PR 2 antagonist JTE-013, suggesting that S1PR 2 impaired insulin signaling. In contrast, ApoM was shown to promote insulin secretion and antagonize insulin resistance after activating S1PR1 and/or S1PR3 signaling (124). The immunomodulator FTY720-phosphate bound to all S1P receptors except S1PR2, but this binding did not reduce insulin signaling (125). The different effects of S1P on insulin resistance were not contradictory, which was mainly related to the binding of S1P with different transporters. S1P played a protective role against insulin resistance by activating insulin signaling pathways such as AKT and AMPK via receptor subtypes S1PR1 and/or S1PR3 (**Figure 2**, **Table 1**), and improved mitochondrial function to promote glucose metabolism in cells, implying that S1P is considered as a new target for screening hypoglycemic agents (126). The interaction of S1P with S1PR2 and peripheral insulin resistance after conversion into Cer could also affect the brain through the pancreatic axis, inducing insulin resistance of neurons and increasing the risk of neurological diseases (**Figure 2**).

### 3.3 Glucosylceramide in BIR

Glucosylceramide (GlcCer) is obtained by adding glucose to Cer scaffold structure, which can be used as the precursor of complex ganglioside. It is also a special sphingolipid type that integrates amino acids, fatty acids and sugars. GlcCer is an independent antagonist of insulin signaling, which is similar to Cer (127). Neuronal activity triggered the synthesis of GlcCer in neurons, which was secreted by neurons via exosomes and subsequently absorbed by glial cells (128). β-glucose ceramidase in lysosomes hydrolyzes GlcCer to glucose and Cer. Subsequently, the generated central and peripheral Cer inhibited AKT insulin pathway via PKCζ or protein phosphatase 2A (**Figure 2**). Ceramide-glucosyltransferase inhibitors have been shown to increase insulin sensitivity and improve glucose tolerance in leptin deficient and diet-induced obesity mice and leptin receptor deficient rats (129, 130). Gangliosides are produced by adding hexose, sialic acid, or hexosamine to the GlcCer structure. Ganglioside GM3 has been shown to impair insulin signaling and block glucose uptake in cell culture studies (127). In agreement, mice lacking GM3 synthase, the enzyme that catalyzes the formation of GM3 ganglioside, showed reduced fasting blood glucose levels and improved glucose tolerance. When challenged with a high-fat diet, GM3 synthase-deficient mice maintained glucose tolerance and increased inhibition of glucose output from the liver (131).

### 3.4 Sphingomyelin in BIR

Sphingomyelin (SM) is one of the most abundant sphingolipids in the body. It is produced by the migration of the phosphocholine portion from phosphatidylcholine to the Cer main chain. SM is hydrolyzed by sphingomyelin enzymes to produce a series of bioactive lipids such as Cer, thus participating in the regulation of downstream pathways. Researchers refered to the dynamic system composed of sphingomyelin, Cer and sphingosine as “sphingomyelin rheostat”, which plays a role in regulating cell survival and proliferation (132). The biosynthesis of SM *in vivo* is mainly dependent on sphingomyelin synthase 1 and 2 (SGMS1/2).

SGMS1-induced golgi-dependent protein kinase D activation negatively regulated Cer transport protein activity by controlling phosphatidylinositol 4-phosphate level, and SGMS2 was required for SM-induced BIR (133). Jiang et al. found that SGMS2 affected the metabolism of lipid and sugar, and could directly regulate sphingomyelin composition of lipid rafts. After SGMS2 gene knockout, the mice not only survived healthy but also resisted atherosclerosis, obesity, fatty liver, T2 DM and insulin resistance caused by a high-fat diet (134). Further evidence of sphingomyelin’s involvement in glucose metabolism disorders also comes from interference with sphingomyelin synthase genes. After ablating the SGMS2 gene in mice fed with a high-fat diet, SM plasma membrane levels and weight gain were reduced, while glucose tolerance and insulin sensitivity were increased (135, 136). Therefore, selective inhibition of SGMS2 is considered as a potential new therapeutic target for metabolic diseases.

### 3.5 Sphingolipid metabolism enzymes involved in BIR

As demonstrated previously, mice with less Cer were less likely to develop insulin resistance (137). Palmitic acid is the substrate for sphingolipids synthesis, and *de novo* synthesis of Cer is controlled by palmitoyl CoA, which activates the rate-limiting enzyme serine-β palmitoyl transferase. Palmitic acid activates serine-β palmitoyl transferase, which is involved in Cer accumulation in astrocytes, enhances cytokine release and activates signaling cascades in neurons (138). C1P is an important metabolite of Cer, which is catalyzed by CERK. Notably, C1P promoted the production of pro-inflammatory eicosanoid (e.g. arachidonic acid), which was a powerful signaling molecule that contributed to chronic inflammation in diseases such as cancer, asthma, atherosclerosis, and thrombosis. In periphery, C1P disrupted the proliferation of adipocyte, forcing them to hypertrophy. Inflammatory signals triggered cell production of C1P from other harmless adipose tissue. When C1P accumulated to a certain level, it inhibited the proliferation of adipocytes, causing them to grow larger and increase insulin resistance (139). Numerous researches have shown that CERK plays an important role in cellular inflammation processes (140, 141). In animal models, CERK^-/-^ mice showed lower blood sugar levels than wild-type mice, suggesting that CERK deficiency can improve insulin sensitivity (142). In a study of Cannabidiol, researchers observed that cannabidiol inactivated GSK-3 in the rats treated with a high-fat diet, leading to increased insulin sensitivity and thus reducing the occurrence of cerebral insulin resistance. The expression of serine palmitoyl transferase 2 and Cer synthase 4 and 6, which are involved in the *de novo* synthesis pathway, was also significantly reduced. These results suggest that the remission of insulin resistance may be indirectly caused by the intervention of cannabidiol on sphingolipid metabolism enzymes (143). In addition, it has been shown that stimulating SMase activity in neurons could cause the subsequent accumulation of ceramide and ROS (two neurotoxic intermediates) (144).

## 4 Discussion

Cognitive impairment occurs as a secondary outcome of DM, and the severity and prevalence of this complication appear to be greater in T2 DM. With the increasing research on the mechanism of sphingolipids in T2 DM, insulin resistance and other human diseases, intervening sphingolipids to improve insulin resistance has become a new strategy for the prevention and treatment of central nervous system dysfunction. Lipid metabolomics studies found that compared with the control group, blood sphingomyelin (145–147), hexose ceramide (94, 148), Cer (145, 146) and other sphingolipids in PD patients significantly changed. In a pathway analysis of the sera of patients with PD, researchers determined that abnormal sphingolipid metabolism induced insulin resistance was the most important pathogenic factor for the disorder (95). An epidemiological meta-analysis reported that lipid-lowering statins were protective against Parkinson’s symptom (149). Furthermore, the incidence of PD was significantly reduced in diabetic patients using certain diabetes drug classes, such as glucagon-like peptide-1 (GLP-1) receptor agonists and GLP-1-degrading enzyme dipeptidyl peptidase 4 inhibitors (150). These studies reveal a link between sphingolipids dysregulation and metabolic pathway function in neurodegenerative diseases including PD, which is of great importance to neurodegenerative diseases therapy. In diseases induced by mutations in sphingolipid metabolism genes, extreme changes in plasma sphingolipids levels may aid in diagnosis, for example, the GlcCer for Gaucher disease (151). However, plasma sphingolipids are less applicable in the diagnosis of sporadic neurodegenerative diseases, such as AD, PD, and Lewy body dementia. The ability of changes in sphingolipids and sphingolipid metabolism enzymes to predict and diagnose occasional diseases of the nervous system remains to be explored, and it is recommended to combine with lipidomics or exosome diagnostic strategies.

At present, the methods to control sphingolipids level mainly focused on the intervention of sphingolipids synthesis. Inhibitors like myriocin, L-cycloserine and C8-cyclopropenylceramide are used to inhibit Cer synthesis, while AMP-DNM and Genz-123346 are used to inhibit glucosylceramide synthase. In recent years, it has become a hot topic to design drugs to treat diseases by targeting enzymes involved in sphingolipid metabolism. The most successful attempt is the S1P agonist, fingolimod (FTY720), a structural analogue of sphingosine that is phosphorylated by SK2 *in vivo*. Its development is attributed to scientists’ exploration of small molecule immunosuppressants. Myriocin, found in the Chinese herb Cordyceps sinensis Sacc, undergoes a series of structural modifications to obtain fingolimod. The drug was approved by the Food and Drug Administration in 2010 and was the first oral formulation approved for the treatment of multiple sclerosis. At the same time, based on the structure and pharmacological action of fingolimod, continuously optimized drug molecules have emerged in an endless stream. Selective activation/inhibition of different S1PR can produce better targeted drugs, such as siponimod, which can selectively bind to the S1PR1 receptor subtype expressed on the surface of lymphocytes, and prevent lymphocytes from entering the central nervous system of MS patients, thereby reducing inflammation. In addition, KRP203 and CS-0777 are also under clinical studies. However, the research and development of drugs treated AD/PD are still controversial and bottleneck. Insulin resistance is the culprit behind T2 DM, nevertheless, it is unlikely that a single factor is the main cause of neurocognitive dysfunction in T2 DM patients. Complex pathomechanism elements such as hyperglycemia, obesity, neuroinflammation, oxidative stress, and Aβ plaques also play a crucial role in cognitive abnormalities individually or synergistically. Abnormal sphingolipid metabolism, as one of the mechanisms of brain insulin resistance, is controversial on the position in the pathogenesis of multiple factors. Therefore, targeting sphingolipid-only strategies may be of limited use in patients with neurological diseases whose pathogenesis is unclear. Although many destructive effects of abnormal sphingolipid metabolism have been known, its etiology and pathophysiology still remain unclear. Studies on the biological effects of sphingolipids and brain insulin resistance are still inconsistent. The mechanism by which they regulate the function of nerve cells is very complex and requires further investigation.

## 5 Summary and prospect

Sphingolipids are a large family of active lipids with important physiological functions. Their biological effects are rich and varied, and they are associated with many diseases such as inflammation, autoimmune diseases, tumors, diabetes and insulin resistance. The signaling pathways that sphingolipids participate in can regulate the production or degradation of sphingolipids or regulate the receptors of sphingolipids, so as to treat and alleviate diseases. Many enzymes involved in sphingolipid synthesis or metabolism have become potential therapeutic targets for a variety of metabolic diseases.

This article reviews the research progress of sphingolipids in insulin resistance, especially in BIR. sphingolipids, not only constitute the structure of plasma membrane, but also directly or indirectly participate in cellular glucose metabolism and insulin resistance through the classical insulin signaling pathway, such as PI3K-AKT-mTOR. Insulin resistance in the brain, where sugar metabolism is unusually active, has been linked to various neurological diseases to varying degrees. In-depth understanding of the mechanism of cerebral insulin resistance is helpful for etiological research and subsequent development of new drugs. Currently, there have been drugs targeting sphingolipids (such as S1PR modulators), or studies targeting palmitoleic acid isomer, the precursor of sphingolipids, entered clinical trials. The study of palmitoleic acid was aimed at enhancing insulin sensitivity (NCT02311790) (**Table 2**). It is believed that with the continuous deepening of research, the mechanism of sphingolipids’ involvement in insulin resistance and the mechanism of brain lesions will become clearer, which will bring new targets and hope for the treatment of diabetes, AD, PD, tumor and other related diseases.

**Table 2:** Progress of clinical trials on sphingolipids and insulin resistance and related diseases.

Study title	Conditions	Status	Year	Reference source
Brain Imaging Study of Rosiglitazone Efficacy and Safety in Alzheimer**’**s Disease	AD	Completed	2005	NCT00265148
Rosiglitazone Effects on Cognition for Adults in Later Life	Mild Cognitive Impairment	Unknown	2005	NCT00242593
Mutations Associated with Parkinson s Disease	PD	Terminated	2012	NCT01547832
Effect of Insulin Sensitizer Metformin on AD Biomarkers	AD, Vascular Dementia, Dementia, Memory Impairment	Completed	2013	NCT01965756
Alzheimer**’**s Disease Biomarkers in Cerebrospinal Fluid in Insulin-resistant Men	AD	Completed	2013	NCT02010476
Palmitoleic Isomer Study	Insulin Sensitivity	Completed	2014	NCT02311790
Macronutrient Effects on Alzheimer**’**s Disease (MEAL-2)	Prediabetic State, Insulin Resistance, Middle Age, (and 2 more…)	Completed	2015	NCT02463084
Intermittent Calorie Restriction, Insulin Resistance, and Biomarkers of Brain Function	AD, Obesity, Diabetes Mellitus	Completed	2015	NCT02460783
BDPP Treatment for Mild Cognitive Impairment (MCI) and Prediabetes or Type 2 Diabetes Mellitus (T2 DM)	Mild Cognitive Impairment, AD	Completed	2015	NCT02502253
Phospholipid and Sphingolipids Composition of High-density Lipoproteins (HDL) in Obese Non-diabetic Patients with Metabolic Syndrome	Metabolic Syndrome	Completed	2016	NCT02851602
Serum Sphingolipidomic Analyses in Healthy, Diabetic and Prediabetic Subjects	Type 2 Diabetes Mellitus, Prediabetic State	Unknown	2016	NCT02826759
Effects of Chromium on Insulin Resistance in Alzheimer Disease Patients	Alzheimer Disease	Unknown	2017	NCT03038282
Skeletal Muscle Diacylglycerol and Sphingolipids-Impact of Localization and Species on Insulin Resistance in Humans	Type 2 Diabetes Mellitus, Pre-diabetes, Obesity	Completed	2017	NCT03077360
Ceramides in Muscle During Insulin Resistance	Insulin Resistance	Withdrawn	2018	NCT03731598
Plasma Dihydroceramides Are Associated with Hepatic Steatosis in Type 1 and Type 2 Diabetes	Type 2 Diabetes Mellitus, Type 1 Diabetes Mellitus	Completed	2018	NCT03447964
Vanderbilt Memory and Aging Project	Alzheimer Disease, Aging, Aged, 80 and Over, (and 5 more…)	Recruiting	2022	NCT05372159

## Author contributions

MM and YL conceived and designed the framework of this review. MM investigated references and wrote the manuscript with assistance of ML and YM. JZ was responsible for the production of charts and tables. YL, JZ and ML revised the manuscript. All authors have read and approved the final manuscript.

## References

[1] Samuel VT Shulman GI. Nonalcoholic fatty liver disease, insulin resistance, and ceramides. N Engl J Med (2019) 381(19):1866–9. doi: 10.1056/NEJMcibr1910023 31693811

[2] Hammerschmidt P Ostkotte D Nolte H Gerl MJ Jais A Brunner HL. CerS6-derived sphingolipids interact with mff and promote mitochondrial fragmentation in obesity. Cell (2019) 177 (6):1536–52. doi: 10.1016/j.cell.2019.05.008 31150623

[3] Chew WS Seow WL Chong JR Lai MKP Torta F Wenk MR. Sphingolipidomics analysis of large clinical cohorts. Part 1: Technical notes and practical considerations. Biochem Biophys Res Commun (2018) 504(3):596–601. doi: 10.1016/j.bbrc.2018.04.076 29654754

[4] Hannun YA Obeid LM. Sphingolipids and their metabolism in physiology and disease. Nat Rev Mol Cell Biol (2018) 19(3):175–91. doi: 10.1038/nrm.2017.107 PMC5902181 29165427

[5] Clarke CJ D'Angelo G Silva LC. Sphingolipid metabolism and signaling: embracing diversity. FEBS Lett (2020) 594(22):3579–82. doi: 10.1002/1873-3468.13979 33241880

[6] Haslam TM Feussner I. Diversity in sphingolipid metabolism across land plants. J Exp Bot (2022) 73(9):2785–98. doi: 10.1093/jxb/erab558 PMC9113257 35560193

[7] Iessi E Marconi M Manganelli V Sorice M Malorni W Garofalo T. On the role of sphingolipids in cell survival and death. Int Rev Cell Mol Biol (2020) 351:149–95. doi: 10.1016/bs.ircmb.2020.02.004 32247579

[8] Ogretmen B. Sphingolipid metabolism in cancer signalling and therapy. Nat Rev Cancer. (2018) 18(1):33–50. doi: 10.1038/nrc.2017.96 29147025 PMC5818153

[9] Damjanovich S Bene L Matkó J Mátyus L Krasznai Z Szabó G. Two-dimensional receptor patterns in the plasma membrane of cells. A critical evaluation of their identification, origin and information content. Biophys Chem (1999) 82 (2-3):99–108 doi: 10.1016/S0301-4622(99)00109-X 17030342

[10] Magal LG Yaffe Y Shepshelovich J Aranda JF de Marco M Gaus K. Clustering and lateral concentration of raft lipids by the MAL protein. Mol Biol Cell (2009) 20(16):3751–62. doi: 10.1091/mbc.e09-02-0142 PMC2777934 19553470

[11] Lin G Wang L Marcogliese PC Bellen HJ. Sphingolipids in the pathogenesis of parkinson's disease and parkinsonism. Trends Endocrinol Metab (2019) 30(2):106–17. doi: 10.1016/j.tem.2018.11.003 30528460

[12] Braun PE Morell P Radin NS. Synthesis of C18- and C20-dihydrosphingosines, ketodihydrosphingosines, and ceramides by microsomal preparations from mouse brain. J Biol Chem (1970) 245(2):335–41. doi: 10.1016/S0021-9258(18)63397-6 4391620

[13] Batheja AD Uhlinger DJ Carton JM Ho G D'Andrea MR. Characterization of serine palmitoyltransferase in normal human tissues. J Histochem Cytochem (2003) 51(5):687–96. doi: 10.1177/002215540305100514 12704216

[14] Merrill AH. Sphingolipid and glycosphingolipid metabolic pathways in the era of sphingolipidomics. Chem Rev (2011) 111(10):6387–422. doi: 10.1021/cr2002917 PMC3191729 21942574

[15] Kitatani K Idkowiak-Baldys J Hannun YA. The sphingolipid salvage pathway in ceramide metabolism and signaling. Cell Signal (2008) 20(6):1010–8. doi: 10.1016/j.cellsig.2007.12.006 PMC2422835 18191382

[16] Sasset L Di Lorenzo A. Sphingolipid metabolism and signaling in endothelial cell functions. Adv Exp Med Biol (2022) 1372:87–117. doi: 10.1007/978-981-19-0394-6_8 PMC12938882 35503177

[17] Vos M Dulovic-Mahlow M Mandik F Frese L Kanana Y Haissatou Diaw S. Ceramide accumulation induces mitophagy and impairs β-oxidation in PINK1 deficiency. Proc Natl Acad Sci U.S.A. (2021) 118 (43):e2025347118. doi: 10.1073/pnas.2025347118 34686591 PMC8639384

[18] Hannun YA Obeid LM. Principles of bioactive lipid signalling: lessons from sphingolipids. Nat Rev Mol Cell Biol (2008) 9(2):139–50. doi: 10.1038/nrm2329 18216770

[19] Hannun YA Bell RM. Lysosphingolipids inhibit protein kinase C: implications for the sphingolipidoses. Science (1987) 235(4789):670–4. doi: 10.1126/science.3101176 3101176

[20] Hadas Y Vincek AS Youssef E Żak MM Chepurko E Sultana N. Altering sphingolipid metabolism attenuates cell death and inflammatory response after myocardial infarction. Circulation (2020) 141(11):916–30. doi: 10.1161/CIRCULATIONAHA.119.041882 PMC7135928 31992066

[21] van Leent MMT Beldman TJ Toner YC Lameijer MA Rother N Bekkering S. Prosaposin mediates inflammation in atherosclerosis. Sci Transl Med (2021) 13 (584):eabe1433. doi: 10.1126/scitranslmed.abe1433 33692130 PMC8209679

[22] Canals D Clarke CJ. Compartmentalization of Sphingolipid metabolism: Implications for signaling and therapy. Pharmacol Ther (2022) 232:108005. doi: 10.1016/j.pharmthera.2021.108005 34582834 PMC9619385

[23] Huang Q Chen C Chen W Cai C Xing H Li J. Cell type- and region-specific translatomes in an MPTP mouse model of Parkinson's disease. Neurobiol Dis (2023) 180:106105. doi: 10.1016/j.nbd.2023.106105 36977454

[24] Yuyama K Sun H Mitsutake S Igarashi Y. Sphingolipid-modulated exosome secretion promotes clearance of amyloid-β by microglia. J Biol Chem (2012) 287(14):10977–89. doi: 10.1074/jbc.M111.324616 PMC3322859 22303002

[25] Czubowicz K Jęśko H Wencel P Lukiw WJ Strosznajder RP. The role of ceramide and sphingosine-1-phosphate in alzheimer's disease and other neurodegenerative disorders. Mol Neurobiol (2019) 56(8):5436–55. doi: 10.1007/s12035-018-1448-3 PMC6614129 30612333

[26] Prinz M Jung S Priller J. Microglia biology: one century of evolving concepts. Cell (2019) 179(2):292–311. doi: 10.1016/j.cell.2019.08.053 31585077

[27] Chu J Yang J Zhou Y Chen J Chen KH Zhang C. ATP-releasing SWELL1 channel in spinal microglia contributes to neuropathic pain. Sci Adv (2023) 9(13):eade9931. doi: 10.1126/sciadv.ade9931 36989353 PMC10058245

[28] Bartels T De Schepper S Hong S. Microglia modulate neurodegeneration in Alzheimer's and Parkinson's diseases. Science (2020) 370(6512):66–9. doi: 10.1126/science.abb8587 33004513

[29] Robak LA Jansen IE van Rooij J Uitterlinden AG Kraaij R Jankovic J. Excessive burden of lysosomal storage disorder gene variants in Parkinson's disease. Brain (2017) 140(12):3191–203. doi: 10.1093/brain/awx285 PMC5841393 29140481

[30] Sidransky E Nalls MA Aasly JO Aharon-Peretz J Annesi G Barbosa ER. Multicenter analysis of glucocerebrosidase mutations in Parkinson's disease. N Engl J Med (2009) 361(17):1651–61. doi: 10.1056/NEJMoa0901281 PMC2856322 19846850

[31] Saunders A Macosko EZ Wysoker A Goldman M Krienen FM de Rivera H. Molecular diversity and specializations among the cells of the adult mouse brain. Cell (2018) 174 (4):1015–1030.e16. doi: 10.1016/j.cell.2018.07.028 PMC6447408 30096299

[32] Wheeler MA Quintana FJ. Regulation of astrocyte functions in multiple sclerosis. Cold Spring Harb Perspect Med (2019) 9 (1):a029009 doi: 10.1101/cshperspect.a029009 29358321 PMC6314073

[33] Chao C-C Gutiérrez-Vázquez C Rothhammer V Mayo L Wheeler MA Tjon EC. Metabolic Control of Astrocyte Pathogenic Activity *via* cPLA2-MAVS. Cell (2019) 179 (7):1483–98. doi: 10.1016/j.cell.2019.11.016 PMC6936326 31813625

[34] Rothhammer V Kenison JE Tjon E Takenaka MC de Lima KA Borucki DM. Sphingosine 1-phosphate receptor modulation suppresses pathogenic astrocyte activation and chronic progressive CNS inflammation. Proc Natl Acad Sci U S A. (2017) 114(8):2012–7. doi: 10.1073/pnas.1615413114 PMC5338419 28167760

[35] Choi JW Gardell SE Herr DR Rivera R Lee C-W Noguchi K. FTY720 (fingolimod) efficacy in an animal model of multiple sclerosis requires astrocyte sphingosine 1-phosphate receptor 1 (S1P1) modulation. Proc Natl Acad Sci U S A. (2011) 108(2):751–6. doi: 10.1073/pnas.1014154108 PMC3021041 21177428

[36] Fabelo N Martín V Marín R Moreno D Ferrer I Díaz M. Altered lipid composition in cortical lipid rafts occurs at early stages of sporadic Alzheimer's disease and facilitates APP/BACE1 interactions. Neurobiol Aging. (2014) 35(8):1801–12. doi: 10.1016/j.neurobiolaging.2014.02.005 24613671

[37] Priller C Bauer T Mitteregger G Krebs B Kretzschmar HA Herms J. Synapse formation and function is modulated by the amyloid precursor protein. J Neurosci (2006) 26(27):7212–21. doi: 10.1523/JNEUROSCI.1450-06.2006 PMC6673945 16822978

[38] Siskind LJ Kolesnick RN Colombini M. Ceramide channels increase the permeability of the mitochondrial outer membrane to small proteins. J Biol Chem (2002) 277(30):26796–803. doi: 10.1074/jbc.M200754200 PMC2246046 12006562

[39] Kogot-Levin A Saada A. Ceramide and the mitochondrial respiratory chain. Biochimie (2014) 100:88–94. doi: 10.1016/j.biochi.2013.07.027 23933096

[40] Novgorodov SA Voltin JR Gooz MA Li L Lemasters JJ Gudz TI. Acid sphingomyelinase promotes mitochondrial dysfunction due to glutamate-induced regulated necrosis. J Lipid Res (2018) 59(2):312–29. doi: 10.1194/jlr.M080374 PMC5794425 29282302

[41] Law BA Liao X Moore KS Southard A Roddy P Ji R. Lipotoxic very-long-chain ceramides cause mitochondrial dysfunction, oxidative stress, and cell death in cardiomyocytes. FASEB J (2018) 32(3):1403–16. doi: 10.1096/fj.201700300R PMC5892719 29127192

[42] Cutler RG Kelly J Storie K Pedersen WA Tammara A Hatanpaa K. Involvement of oxidative stress-induced abnormalities in ceramide and cholesterol metabolism in brain aging and Alzheimer's disease. Proc Natl Acad Sci U S A. (2004) 101(7):2070–5. doi: 10.1073/pnas.0305799101 PMC357053 14970312

[43] Kornfeld S. Trafficking of lysosomal enzymes. FASEB J (1987) 1(6):462–8. doi: 10.1096/fasebj.1.6.3315809 3315809

[44] van Echten-Deckert G Walter J. Sphingolipids: critical players in Alzheimer's disease. Prog Lipid Res (2012) 51(4):378–93. doi: 10.1016/j.plipres.2012.07.001 22835784

[45] Taguchi YV Liu J Ruan J Pacheco J Zhang X Abbasi J. Glucosylsphingosine promotes α-synuclein pathology in mutant GBA-associated parkinson's disease. J Neurosci (2017) 37(40):9617–31. doi: 10.1523/JNEUROSCI.1525-17.2017 PMC5628407 28847804

[46] Yang SN. Ceramide-induced sustained depression of synaptic currents mediated by ionotropic glutamate receptors in the hippocampus: an essential role of postsynaptic protein phosphatases. Neuroscience (2000) 96(2):253–8. doi: 10.1016/S0306-4522(99)00582-5 10683565

[47] Huwiler A Zangemeister-Wittke U. The sphingosine 1-phosphate receptor modulator fingolimod as a therapeutic agent: Recent findings and new perspectives. Pharmacol Ther (2018) 185:34–49. doi: 10.1016/j.pharmthera.2017.11.001 29127024

[48] Das A Arifuzzaman S Kim SH Lee YS Jung KH Chai YG. FTY720 (fingolimod) regulates key target genes essential for inflammation in microglial cells as defined by high-resolution mRNA sequencing. Neuropharmacology (2017) 119:1–14. doi: 10.1016/j.neuropharm.2017.03.034 28373076

[49] Foster CA Mechtcheriakova D Storch MK Balatoni B Howard LM Bornancin F. FTY720 rescue therapy in the dark agouti rat model of experimental autoimmune encephalomyelitis: expression of central nervous system genes and reversal of blood-brain-barrier damage. Brain Pathol (2009) 19(2):254–66. doi: 10.1111/j.1750-3639.2008.00182.x PMC8094834 18540945

[50] Magistretti PJ Allaman I. Lactate in the brain: from metabolic end-product to signalling molecule. Nat Rev Neurosci (2018) 19(4):235–49. doi: 10.1038/nrn.2018.19 29515192

[51] Mergenthaler P Lindauer U Dienel GA Meisel A. Sugar for the brain: the role of glucose in physiological and pathological brain function. Trends Neurosci (2013) 36(10):587–97. doi: 10.1016/j.tins.2013.07.001 PMC3900881 23968694

[52] Dienel GA. Physiol Rev (2019) 99 (1):949–1045. doi: 10.1152/physrev.00062.2017 30565508

[53] Camandola S Mattson MP. Brain metabolism in health, aging, and neurodegeneration. EMBO J (2017) 36(11):1474–92. doi: 10.15252/embj.201695810 PMC5452017 28438892

[54] Cunnane SC Courchesne-Loyer A Vandenberghe C St-Pierre V Fortier M Hennebelle M. Can ketones help rescue brain fuel supply in later life? Implications for cognitive health during aging and the treatment of alzheimer's disease. Front Mol Neurosci (2016) 9:53. doi: 10.3389/fnmol.2016.00053 27458340 PMC4937039

[55] Zilberter Y Zilberter M. The vicious circle of hypometabolism in neurodegenerative diseases: Ways and mechanisms of metabolic correction. J Neurosci Res (2017) 95(11):2217–35. doi: 10.1002/jnr.24064 28463438

[56] Calvo-Ochoa E Arias C. Cellular and metabolic alterations in the hippocampus caused by insulin signalling dysfunction and its association with cognitive impairment during aging and Alzheimer's disease: studies in animal models. Diabetes Metab Res Rev (2015) 31 (1):1–13. doi: 10.1002/dmrr.2531 24464982

[57] Scapin G Dandey VP Zhang Z Prosise W Hruza A Kelly T. Structure of the insulin receptor-insulin complex by single-particle cryo-EM analysis. Nature (2018) 556(7699):122–5. doi: 10.1038/nature26153 PMC5886813 29512653

[58] Chen W Cai W Hoover B Kahn CR. Insulin action in the brain: cell types, circuits, and diseases. Trends Neurosci (2022) 45(5):384–400. doi: 10.1016/j.tins.2022.03.001 35361499 PMC9035105

[59] Kullmann S Hummel J Wagner R Dannecker C Vosseler A Fritsche L. Empagliflozin improves insulin sensitivity of the hypothalamus in humans with prediabetes: A randomized, double-blind, placebo-controlled, phase 2 trial. Diabetes Care (2022) 45(2):398–406. doi: 10.2337/dc21-1136 34716213 PMC8914418

[60] Reaven GM. The insulin resistance syndrome: definition and dietary approaches to treatment. Annu Rev Nutr (2005) 25:391–406. doi: 10.1146/annurev.nutr.24.012003.132155 16011472

[61] Sachdeva AK Dharavath RN Chopra K. Time-response studies on development of cognitive deficits in an experimental model of insulin resistance. Clin Nutr (2019) 38(3):1447–56. doi: 10.1016/j.clnu.2018.06.966 30037709

[62] Roep BO Thomaidou S van Tienhoven R Zaldumbide A. Type 1 diabetes mellitus as a disease of the β-cell (do not blame the immune system?). Nat Rev Endocrinol (2021) 17(3):150–61. doi: 10.1038/s41574-020-00443-4 PMC7722981 33293704

[63] Galicia-Garcia U Benito-Vicente A Jebari S Larrea-Sebal A Siddiqi H Uribe KB. Pathophysiology of type 2 diabetes mellitus. Int J Mol Sci (2020) 21 (17):6275. doi: 10.3390/ijms21176275 PMC7503727 32872570

[64] Brundel M Kappelle LJ Biessels GJ. Brain imaging in type 2 diabetes. Eur Neuropsychopharmacol (2014) 24(12):1967–81. doi: 10.1016/j.euroneuro.2014.01.023 24726582

[65] Del Bene A Ciolli L Borgheresi L Poggesi A Inzitari D Pantoni L. Is type 2 diabetes related to leukoaraiosis? an updated review. Acta Neurol Scand (2015) 132(3):147–55. doi: 10.1111/ane.12398 25772411

[66] Arnold SE Lucki I Brookshire BR Carlson GC Browne CA Kazi H. High fat diet produces brain insulin resistance, synaptodendritic abnormalities and altered behavior in mice. Neurobiol Dis (2014) 67:79–87. doi: 10.1016/j.nbd.2014.03.011 24686304 PMC4083060

[67] Martins IVA Rivers-Auty J Allan SM Lawrence CB. Mitochondrial abnormalities and synaptic loss underlie memory deficits seen in mouse models of obesity and alzheimer's disease. J Alzheimers Dis (2017) 55(3):915–32. doi: 10.3233/JAD-160640 PMC5278950 27802235

[68] Novak V Milberg W Hao Y Munshi M Novak P Galica A. Enhancement of vasoreactivity and cognition by intranasal insulin in type 2 diabetes. Diabetes Care (2014) 37(3):751–9. doi: 10.2337/dc13-1672 PMC3931384 24101698

[69] Zhang H Hao Y Manor B Novak P Milberg W Zhang J. Intranasal insulin enhanced resting-state functional connectivity of hippocampal regions in type 2 diabetes. Diabetes (2015) 64(3):1025–34. doi: 10.2337/db14-1000 PMC4338591 25249577

[70] Chornenkyy Y Wang W-X Wei A Nelson PT. Alzheimer's disease and type 2 diabetes mellitus are distinct diseases with potential overlapping metabolic dysfunction upstream of observed cognitive decline. Brain Pathol (2019) 29 (1):3–17. doi: 10.1111/bpa.12655 PMC6427919 30106209

[71] Steen E Terry BM Rivera EJ Cannon JL Neely TR Tavares R. Impaired insulin and insulin-like growth factor expression and signaling mechanisms in Alzheimer's disease–is this type 3 diabetes? J Alzheimers Dis (2005) 7(1):63–80. doi: 10.3233/JAD-2005-7107 15750215

[72] Arnold SE Arvanitakis Z Macauley-Rambach SL Koenig AM Wang H-Y Ahima RS. Brain insulin resistance in type 2 diabetes and Alzheimer disease: concepts and conundrums. Nat Rev Neurol (2018) 14(3):168–81. doi: 10.1038/nrneurol.2017.185 PMC6098968 29377010

[73] Zhang Y Huang N-Q Yan F Jin H Zhou S-Y Shi J-S. Diabetes mellitus and Alzheimer's disease: GSK-3β as a potential link. Behav Brain Res (2018) 339:57–65. doi: 10.1016/j.bbr.2017.11.015 29158110

[74] Fang EF Hou Y Palikaras K Adriaanse BA Kerr JS Yang B. Mitophagy inhibits amyloid-β and tau pathology and reverses cognitive deficits in models of Alzheimer's disease. Nat Neurosci (2019) 22(3):401–12. doi: 10.1038/s41593-018-0332-9 PMC6693625 30742114

[75] Tong M Dong M de la Monte SM. Brain insulin-like growth factor and neurotrophin resistance in Parkinson's disease and dementia with Lewy bodies: potential role of manganese neurotoxicity. J Alzheimers Dis (2009) 16(3):585–99. doi: 10.3233/JAD-2009-0995 PMC2852260 19276553

[76] Kakoty V Kc S Kumari S Yang C-H Dubey SK Sahebkar A. Brain insulin resistance linked Alzheimer's and Parkinson's disease pathology: An undying implication of epigenetic and autophagy modulation. Inflammopharmacology (2023). 31(2):699–716. doi: 10.1007/s10787-023-01187-z 36952096

[77] Duarte AI Candeias E Correia SC Santos RX Carvalho C Cardoso S. Crosstalk between diabetes and brain: glucagon-like peptide-1 mimetics as a promising therapy against neurodegeneration. Biochim Biophys Acta (2013) 1832(4):527–41. doi: 10.1016/j.bbadis.2013.01.008 23314196

[78] Kleinridders A Cai W Cappellucci L Ghazarian A Collins WR Vienberg SG. Insulin resistance in brain alters dopamine turnover and causes behavioral disorders. Proc Natl Acad Sci U S A. (2015) 112(11):3463–8. doi: 10.1073/pnas.1500877112 PMC4371978 25733901

[79] Perruolo G Viggiano D Fiory F Cassese A Nigro C Liotti A. Parkinson-like phenotype in insulin-resistant PED/PEA-15 transgenic mice. Sci Rep (2016) 6:29967. doi: 10.1038/srep29967 27426254 PMC4947959

[80] Schapira AHV Olanow CW Greenamyre JT Bezard E. Slowing of neurodegeneration in Parkinson's disease and Huntington's disease: future therapeutic perspectives. Lancet (2014) 384(9942):545–55. doi: 10.1016/S0140-6736(14)61010-2 24954676

[81] Kim B Feldman EL. Insulin resistance in the nervous system. Trends Endocrinol Metab (2012) 23(3):133–41. doi: 10.1016/j.tem.2011.12.004 PMC3392648 22245457

[82] Couttas TA Kain N Daniels B Lim XY Shepherd C Kril J. Loss of the neuroprotective factor Sphingosine 1-phosphate early in Alzheimer's disease pathogenesis. Acta Neuropathol Commun (2014) 2:9. doi: 10.1186/2051-5960-2-9 24456642 PMC3906863

[83] Filippov V Song MA Zhang K Vinters HV Tung S Kirsch WM. Increased ceramide in brains with Alzheimer's and other neurodegenerative diseases. J Alzheimers Dis (2012) 29(3):537–47. doi: 10.3233/JAD-2011-111202 PMC3643694 22258513

[84] Han X Rozen S Boyle SH Hellegers C Cheng H Burke JR. Metabolomics in early Alzheimer's disease: identification of altered plasma sphingolipidome using shotgun lipidomics. PloS One (2011) 6(7):e21643. doi: 10.1371/journal.pone.0021643 21779331 PMC3136924

[85] Savica R Murray ME Persson X-M Kantarci K Parisi JE Dickson DW. Plasma sphingolipid changes with autopsy-confirmed Lewy Body or Alzheimer's pathology. Alzheimers Dement (Amst). (2016) 3:43–50. doi: 10.1016/j.dadm.2016.02.005 27152320 PMC4852484

[86] Ellis B Hye A Snowden SG. Metabolic modifications in human biofluids suggest the involvement of sphingolipid, antioxidant, and glutamate metabolism in alzheimer's disease pathogenesis. J Alzheimers Dis (2015) 46(2):313–27. doi: 10.3233/JAD-141899 25835424

[87] Caughlin S Maheshwari S Agca Y Agca C Harris AJ Jurcic K. Membrane-lipid homeostasis in a prodromal rat model of Alzheimer's disease: Characteristic profiles in ganglioside distributions during aging detected using MALDI imaging mass spectrometry. Biochim Biophys Acta Gen Subj. (2018) 1862(6):1327–38. doi: 10.1016/j.bbagen.2018.03.011 29545134

[88] Kaya I Brinet D Michno W Syvänen S Sehlin D Zetterberg H. Delineating amyloid plaque associated neuronal sphingolipids in transgenic alzheimer's disease mice (tgArcSwe) using MALDI imaging mass spectrometry. ACS Chem Neurosci (2017) 8(2):347–55. doi: 10.1021/acschemneuro.6b00391 PMC5314428 27984697

[89] Barrier L Ingrand S Damjanac M Rioux Bilan A Hugon J Page G. Genotype-related changes of ganglioside composition in brain regions of transgenic mouse models of Alzheimer's disease. Neurobiol Aging. (2007) 28(12):1863–72. doi: 10.1016/j.neurobiolaging.2006.08.002 17007963

[90] Chan RB Oliveira TG Cortes EP Honig LS Duff KE Small SA. Comparative lipidomic analysis of mouse and human brain with Alzheimer disease. J Biol Chem (2012) 287(4):2678–88. doi: 10.1074/jbc.M111.274142 PMC3268426 22134919

[91] Katsel P Li C Haroutunian V. Gene expression alterations in the sphingolipid metabolism pathways during progression of dementia and Alzheimer's disease: a shift toward ceramide accumulation at the earliest recognizable stages of Alzheimer's disease? Neurochem Res (2007) 32(4-5):845–56. doi: 10.1007/s11064-007-9297-x 17342407

[92] He X Huang Y Li B Gong C-X Schuchman EH. Deregulation of sphingolipid metabolism in Alzheimer's disease. Neurobiol Aging. (2010) 31(3):398–408. doi: 10.1016/j.neurobiolaging.2008.05.010 18547682 PMC2829762

[93] Grimm MOW Hundsdörfer B Grösgen S Mett J Zimmer VC Stahlmann CP. PS dependent APP cleavage regulates glucosylceramide synthase and is affected in Alzheimer's disease. Cell Physiol Biochem (2014) 34 (1):92–110. doi: 10.1159/000362987 24977484

[94] Hu L Dong M-X Huang Y-L Lu C-Q Qian Q Zhang C-C. Integrated metabolomics and proteomics analysis reveals plasma lipid metabolic disturbance in patients with parkinson's disease. Front Mol Neurosci (2020) 13:80. doi: 10.3389/fnmol.2020.00080 32714143 PMC7344253

[95] Galper J Dean NJ Pickford R Lewis SJG Halliday GM Kim WS. Lipid pathway dysfunction is prevalent in patients with Parkinson's disease. Brain (2022) 145(10):3472–87. doi: 10.1093/brain/awac176 PMC9586542 35551349

[96] Sivasubramanian M Kanagaraj N Dheen ST Tay SSW. Sphingosine kinase 2 and sphingosine-1-phosphate promotes mitochondrial function in dopaminergic neurons of mouse model of Parkinson's disease and in MPP+ -treated MN9D cells in vitro. Neuroscience (2015) 290:636–48. doi: 10.1016/j.neuroscience.2015.01.032 25637806

[97] Pyszko JA Strosznajder JB. The key role of sphingosine kinases in the molecular mechanism of neuronal cell survival and death in an experimental model of Parkinson's disease. Folia Neuropathol. (2014) 52(3):260–9. doi: 10.5114/fn.2014.45567 25310737

[98] Cataldi S Arcuri C Hunot S Légeron F-P Mecca C Garcia-Gil M. Neutral sphingomyelinase behaviour in hippocampus neuroinflammation of MPTP-induced mouse model of parkinson's disease and in embryonic hippocampal cells. Mediators Inflamm (2017) 2017:2470950. doi: 10.1155/2017/2470950 29343884 PMC5733979

[99] Hu F Xu Y Liu F. Hypothalamic roles of mTOR complex I: integration of nutrient and hormone signals to regulate energy homeostasis. Am J Physiol Endocrinol Metab (2016) 310 (11):E994-E1002. doi: 10.1152/ajpendo.00121.2016 27166282 PMC4935144

[100] Zhang Z-Y Dodd GT Tiganis T. Protein tyrosine phosphatases in hypothalamic insulin and leptin signaling. Trends Pharmacol Sci (2015) 36(10):661–74. doi: 10.1016/j.tips.2015.07.003 PMC12831973 26435211

[101] Petersen MC Shulman GI. Mechanisms of insulin action and insulin resistance. Physiol Rev (2018) 98(4):2133–223. doi: 10.1152/physrev.00063.2017 PMC6170977 30067154

[102] Lyn-Cook LE Lawton M Tong M Silbermann E Longato L Jiao P. Hepatic ceramide may mediate brain insulin resistance and neurodegeneration in type 2 diabetes and non-alcoholic steatohepatitis. J Alzheimers Dis (2009) 16(4):715–29. doi: 10.3233/JAD-2009-0984 PMC2893047 19387108

[103] Wigger D Schumacher F Schneider-Schaulies S Kleuser B. Sphingosine 1-phosphate metabolism and insulin signaling. Cell Signal (2021) 82:109959. doi: 10.1016/j.cellsig.2021.109959 33631318

[104] Sánchez-Alegría K Arias C. Functional consequences of brain exposure to saturated fatty acids: From energy metabolism and insulin resistance to neuronal damage. Endocrinol Diabetes Metab (2023) 6(1):e386. doi: 10.1002/edm2.386 36321333 PMC9836261

[105] Dutta BJ Singh S Seksaria S Das Gupta G Singh A. Inside the diabetic brain: Insulin resistance and molecular mechanism associated with cognitive impairment and its possible therapeutic strategies. Pharmacol Res (2022) 182:106358. doi: 10.1016/j.phrs.2022.106358 35863719

[106] Chavez JA Summers SA. A ceramide-centric view of insulin resistance. Cell Metab (2012) 15(5):585–94. doi: 10.1016/j.cmet.2012.04.002 22560211

[107] Holland WL Summers SA. Sphingolipids, insulin resistance, and metabolic disease: new insights from in vivo manipulation of sphingolipid metabolism. Endocrine Rev (2008) 29(4):381–402. doi: 10.1210/er.2007-0025 18451260 PMC2528849

[108] Haus JM Kashyap SR Kasumov T Zhang R Kelly KR DeFronzo RA. Plasma ceramides are elevated in obese subjects with type 2 diabetes and correlate with the severity of insulin resistance. Diabetes (2009) 58(2):337–43. doi: 10.2337/db08-1228 PMC2628606 19008343

[109] Maciejczyk M Żebrowska E Nesterowicz M Supruniuk E Choromańska B Chabowski A. α-lipoic acid reduces ceramide synthesis and neuroinflammation in the hypothalamus of insulin-resistant rats, while in the cerebral cortex diminishes the β-amyloid accumulation. J Inflammation Res (2022) 15:2295–312. doi: 10.2147/JIR.S358799 PMC9005076 35422650

[110] Schwedhelm E Englisch C Niemann L Lezius S von Lucadou M Marmann K. Sphingosine-1-phosphate, motor severity, and progression in parkinson's disease (MARK-PD). Mov Disord (2021) 36(9):2178–82. doi: 10.1002/mds.28652 34008894

[111] Kabayama K Sato T Kitamura F Uemura S Kang BW Igarashi Y. TNFalpha-induced insulin resistance in adipocytes as a membrane microdomain disorder: involvement of ganglioside GM3. Glycobiology (2005) 15(1):21–9. doi: 10.1093/glycob/cwh135 15306563

[112] Kracun I Rosner H Drnovsek V Heffer-Lauc M Cosović C Lauc G. Human brain gangliosides in development, aging and disease. Int J Dev Biol (1991) 35(3):289–95. 1814411

[113] Wu G Lu Z-H Kulkarni N Ledeen RW. Deficiency of ganglioside GM1 correlates with Parkinson's disease in mice and humans. J Neurosci Res (2012) 90(10):1997–2008. doi: 10.1002/jnr.23090 22714832

[114] Ruud J Steculorum SM Brüning JC. Neuronal control of peripheral insulin sensitivity and glucose metabolism. Nat Commun (2017) 8:15259. doi: 10.1038/ncomms15259 28469281 PMC5418592

[115] Chow H-M Shi M Cheng A Gao Y Chen G Song X. Age-related hyperinsulinemia leads to insulin resistance in neurons and cell-cycle-induced senescence. Nat Neurosci (2019) 22(11):1806–19. doi: 10.1038/s41593-019-0505-1 31636448

[116] Kullmann S Kleinridders A Small DM Fritsche A Häring H-U Preissl H. Central nervous pathways of insulin action in the control of metabolism and food intake. Lancet Diabetes Endocrinol (2020) 8(6):524–34. doi: 10.1016/S2213-8587(20)30113-3 32445739

[117] Yang G Badeanlou L Bielawski J Roberts AJ Hannun YA Samad F. Central role of ceramide biosynthesis in body weight regulation, energy metabolism, and the metabolic syndrome. Am J Physiol Endocrinol Metab (2009) 297(1):E211–E24. doi: 10.1152/ajpendo.91014.2008 PMC2711669 19435851

[118] Spinelli M Fusco S Grassi C. Brain insulin resistance and hippocampal plasticity: mechanisms and biomarkers of cognitive decline. Front Neurosci (2019) 13:788. doi: 10.3389/fnins.2019.00788 31417349 PMC6685093

[119] Grillo CA Piroli GG Hendry RM Reagan LP. Insulin-stimulated translocation of GLUT4 to the plasma membrane in rat hippocampus is PI3-kinase dependent. Brain Res (2009) 1296:35–45. doi: 10.1016/j.brainres.2009.08.005 19679110 PMC2997526

[120] Holland WL Brozinick JT Wang L-P Hawkins ED Sargent KM Liu Y. Inhibition of ceramide synthesis ameliorates glucocorticoid-, saturated-fat-, and obesity-induced insulin resistance. Cell Metab (2007) 5(3):167–79. doi: 10.1016/j.cmet.2007.01.002 17339025

[121] Khan MA Khan ZA Shoeb F Fatima G Khan RH Khan MM. Role of de novo lipogenesis in inflammation and insulin resistance in Alzheimer's disease. Int J Biol Macromol (2023) 242(Pt 2):124859. doi: 10.1016/j.ijbiomac.2023.124859 37187418

[122] Afsar SY Alam S Fernandez Gonzalez C van Echten-Deckert G. Sphingosine-1-phosphate-lyase deficiency affects glucose metabolism in a way that abets oncogenesis. Mol Oncol (2022) 16(20):3642–53. doi: 10.1002/1878-0261.13300 PMC9580888 35973936

[123] Yuan Y Jia G Wu C Wang W Cheng L Li Q. Structures of signaling complexes of lipid receptors S1PR1 and S1PR5 reveal mechanisms of activation and drug recognition. Cell Res (2021) 31(12):1263–74. doi: 10.1038/s41422-021-00566-x PMC8441948 34526663

[124] Kurano M Tsukamoto K Shimizu T Kassai H Nakao K Aiba A. Protection against insulin resistance by apolipoprotein M/sphingosine-1-phosphate. Diabetes (2020) 69(5):867–81. doi: 10.2337/db19-0811 31915150

[125] Fayyaz S Henkel J Japtok L Krämer S Damm G Seehofer D. Involvement of sphingosine 1-phosphate in palmitate-induced insulin resistance of hepatocytes *via* the S1P2 receptor subtype. Diabetologia (2014) 57(2):373–82. doi: 10.1007/s00125-013-3123-6 24292566

[126] Fang H Feng Q Shi Y Zhou J Wang Q Zhong L. Hepatic insulin resistance induced by mitochondrial oxidative stress can be ameliorated by sphingosine 1-phosphate. Mol Cell Endocrinol (2020) 501:110660. doi: 10.1016/j.mce.2019.110660 31759099

[127] Chavez JA Siddique MM Wang ST Ching J Shayman JA Summers SA. Ceramides and glucosylceramides are independent antagonists of insulin signaling. J Biol Chem (2014) 289(2):723–34. doi: 10.1074/jbc.M113.522847 PMC3887200 24214972

[128] Wang L Lin G Zuo Z Li Y Byeon SK Pandey A. Neuronal activity induces glucosylceramide that is secreted via exosomes for lysosomal degradation in glia. Sci Adv (2022) 8(28):eabn3326. doi: 10.1126/sciadv.abn3326 35857503 PMC9278864

[129] Aerts JM Ottenhoff R Powlson AS Grefhorst A van Eijk M Dubbelhuis PF. Pharmacological inhibition of glucosylceramide synthase enhances insulin sensitivity. Diabetes (2007) 56(5):1341–9. doi: 10.2337/db06-1619 PMC4298701 17287460

[130] Zhao H Przybylska M Wu IH Zhang J Siegel C Komarnitsky S. Inhibiting glycosphingolipid synthesis improves glycemic control and insulin sensitivity in animal models of type 2 diabetes. Diabetes (2007) 56(5):1210–8. doi: 10.2337/db06-0719 17470562

[131] Yamashita T Hashiramoto A Haluzik M Mizukami H Beck S Norton A. Enhanced insulin sensitivity in mice lacking ganglioside GM3. Proc Natl Acad Sci U S A. (2003) 100(6):3445–9. doi: 10.1073/pnas.0635898100 PMC152312 12629211

[132] Ghasemi R Dargahi L Ahmadiani A. Integrated sphingosine-1 phosphate signaling in the central nervous system: From physiological equilibrium to pathological damage. Pharmacol Res (2016) 104:156–64. doi: 10.1016/j.phrs.2015.11.006 26772814

[133] Kim Y-J Greimel P Hirabayashi Y. GPRC5B-mediated sphingomyelin synthase 2 phosphorylation plays a critical role in insulin resistance. iScience (2018) 8:250–66. doi: 10.1016/j.isci.2018.10.001 PMC6197706 30343189

[134] Mo M Yang J Jiang X-C Cao Y Fei J Chen Y. Discovery of 4-Benzyloxybenzo[ d]isoxazole-3-amine Derivatives as Highly Selective and Orally Efficacious Human Sphingomyelin Synthase 2 Inhibitors that Reduce Chronic Inflammation in db/ db Mice. J Med Chem (2018) 61(18):8241–54. doi: 10.1021/acs.jmedchem.8b00727 30074791

[135] Li Z Zhang H Liu J Liang C-P Li Y Li Y. Reducing plasma membrane sphingomyelin increases insulin sensitivity. Mol Cell Biol (2011) 31(20):4205–18. doi: 10.1128/MCB.05893-11 PMC3187286 21844222

[136] Sugimoto M Shimizu Y Zhao S Ukon N Nishijima K-i Wakabayashi M. Characterization of the role of sphingomyelin synthase 2 in glucose metabolism in whole-body and peripheral tissues in mice. Biochim Biophys Acta (2016) 1861(8 Pt A):688–702. doi: 10.1016/j.bbalip.2016.04.019 27151272

[137] Chaurasia B Kaddai VA Lancaster GI Henstridge DC Sriram S Galam DLA. Adipocyte ceramides regulate subcutaneous adipose browning, inflammation, and metabolism. Cell Metab (2016) 24(6):820–34. doi: 10.1016/j.cmet.2016.10.002 27818258

[138] Liu L Martin R Chan C. Palmitate-activated astrocytes *via* serine palmitoyltransferase increase BACE1 in primary neurons by sphingomyelinases. Neurobiol Aging. (2013) 34(2):540–50. doi: 10.1016/j.neurobiolaging.2012.05.017 PMC3459302 22727944

[139] Yuyama K Mitsutake S Igarashi Y. Pathological roles of ceramide and its metabolites in metabolic syndrome and Alzheimer's disease. Biochim Biophys Acta (2014) 1841(5):793–8. doi: 10.1016/j.bbalip.2013.08.002 23948264

[140] Sugiura M Kono K Liu H Shimizugawa T Minekura H Spiegel S. Ceramide kinase, a novel lipid kinase. Molecular cloning and functional characterization. J Biol Chem (2002) 277(26):23294–300. doi: 10.1074/jbc.M201535200 11956206

[141] Bornancin F. Ceramide kinase: the first decade. Cell Signal (2011) 23 (6):999–1008. doi: 10.1016/j.cellsig.2010.11.012 21111813

[142] Mitsutake S Date T Yokota H Sugiura M Kohama T Igarashi Y. Ceramide kinase deficiency improves diet-induced obesity and insulin resistance. FEBS Lett (2012) 586(9):1300–5. doi: 10.1016/j.febslet.2012.03.032 22465662

[143] Charytoniuk T Sztolsztener K Harasim-Symbor E Berk K Chabowski A Konstantynowicz-Nowicka K. Cannabidiol - A phytocannabinoid that widely affects sphingolipid metabolism under conditions of brain insulin resistance. Biomedicine Pharmacother = Biomed Pharmacotherapie. (2021) 142:112057. doi: 10.1016/j.biopha.2021.112057 34435590

[144] Barth BM Gustafson SJ Kuhn TB. Neutral sphingomyelinase activation precedes NADPH oxidase-dependent damage in neurons exposed to the proinflammatory cytokine tumor necrosis factor-α. J Neurosci Res (2012) 90(1):229–42. doi: 10.1002/jnr.22748 PMC3218197 21932365

[145] Chan RB Perotte AJ Zhou B Liong C Shorr EJ Marder KS. Elevated GM3 plasma concentration in idiopathic Parkinson's disease: A lipidomic analysis. PloS One (2017) 12(2):e0172348. doi: 10.1371/journal.pone.0172348 28212433 PMC5315374

[146] Zhang J Zhang X Wang L Yang C. High performance liquid chromatography-mass spectrometry (LC-MS) based quantitative lipidomics study of ganglioside-NANA-3 plasma to establish its association with parkinson's disease patients. Med Sci Monitor Int Med J Exp Clin Res (2017) 23:5345–53. doi: 10.12659/MSM.904399 PMC5694191 29123078

[147] Stoessel D Schulte C Teixeira Dos Santos MC Scheller D Rebollo-Mesa I Deuschle C. Promising metabolite profiles in the plasma and CSF of early clinical parkinson's disease. Front Aging Neurosci (2018) 10:51. doi: 10.3389/fnagi.2018.00051 29556190 PMC5844983

[148] Klatt-Schreiner K Valek L Kang J-S Khlebtovsky A Trautmann S Hahnefeld L. High glucosylceramides and low anandamide contribute to sensory loss and pain in parkinson's disease. Mov Disord (2020) 35(10):1822–33. doi: 10.1002/mds.28186 32652698

[149] Poly TN Islam MM Walther BA Yang H-C Nguyen P-A Huang C-W. Exploring the association between statin use and the risk of parkinson's disease: A meta-analysis of observational studies. Neuroepidemiology (2017) 49(3-4):142–51. doi: 10.1159/000480401 29145202

[150] Brauer R Wei L Ma T Athauda D Girges C Vijiaratnam N. Diabetes medications and risk of Parkinson's disease: a cohort study of patients with diabetes. Brain (2020) 143(10):3067–76. doi: 10.1093/brain/awaa262 PMC7794498 33011770

[151] van Kruining D Luo Q van Echten-Deckert G Mielke MM Bowman A Ellis S. Sphingolipids as prognostic biomarkers of neurodegeneration, neuroinflammation, and psychiatric diseases and their emerging role in lipidomic investigation methods. Advanced Drug Delivery Rev (2020) 159:232–44. doi: 10.1016/j.addr.2020.04.009 PMC7665829 32360155

